# Transcription Factor Networks Drive Tumor Progression and Immune Microenvironment Remodeling in Hepatocellular Carcinoma

**DOI:** 10.3390/cancers17233787

**Published:** 2025-11-26

**Authors:** Sang Hoon Lee, Ju Won Ahn, Wonbin Choi, Jina Kim, Joon Yeon Hwang, Jae-Hwan Kim, Hyaekang Kim, Woori Kwak

**Affiliations:** 1 Department of Digital Health, Samsung Advanced Institute for Health Sciences & Technology, Sungkyunkwan University, Seoul 06355, Republic of Korea; 2 Department of Medical and Biological Sciences, The Catholic University of Korea, Bucheon 14662, Republic of Korea; 3 Department of Biomedical Science, College of Life Science, CHA University, Seongnam 13488, Republic of Korea; 4 Department of Biotechnology, The Catholic University of Korea, Bucheon 14662, Republic of Korea; 5 Department of Health Sciences and Technology, Samsung Advanced Institute for Health Sciences & Technology, Sungkyunkwan University, Seoul 06355, Republic of Korea; 6 Bio-Resources Bank Division, Nakdonggang National Institute of Biological Resources, Sangju 37242, Republic of Korea

**Keywords:** hepatocellular carcinoma (HCC), tumor microenvironment (TME), single-cell RNA-seq, transcription factors, SPP1, macrophages, immune evasion, cancer progression

## Abstract

(1) Background: Hepatocellular carcinoma (HCC) remains a major cause of cancer mortality and is characterized by pronounced inter- and intra-tumoral heterogeneity and therapy resistance. We aimed to define core transcriptional circuits that drive HCC malignancy and to delineate how these programs shape the tumor microenvironment (TME). (2) Methods: We integrated single-cell, spatial, and bulk transcriptomic datasets from public cohorts. (3) Results: We identified nine tumor-restricted transcription factors (TFs)—HTATIP2, HES6, ILF2, E2F1, MYBL2, DDIT3, FOXM1, HMGA1, and ETV4—whose expression and regulon activity associated with malignant phenotypes and poor survival. These TFs organize a progression axis from an early proliferative state (cluster C4) toward an invasive, metabolically adapted state (cluster C1) enriched for hypoxia, epithelial–mesenchymal transition (EMT), and inflammatory signaling. The C1 state remodeled the TME by establishing an immunosuppressive niche marked by expansion of T regulatory cells (Treg) and by accumulation of SPP1^+^ macrophages. These macrophages, recruited and polarized by C1 tumor cells, exhibited M2-like, pro-angiogenic, and immunosuppressive features and engaged epithelial, immune, and stromal partners via SPP1-CD44 and SPP1-integrin interactions. (4) Conclusions: In summary, a tumor-intrinsic TF network cooperates with SPP1^+^ macrophage signaling to promote a permissive microenvironment and HCC progression. This integrated axis highlights tractable vulnerabilities for therapeutic intervention.

## Simple Summary

Hepatocellular carcinoma (HCC) is a deadly and highly complex cancer. To better understand what makes it aggressive, we investigated the key genetic switches, known as transcription factors (TFs), that control tumor cell behavior. By analyzing vast amounts of genetic data from single cells, we identified a core network of nine TFs that drive HCC progression. We found that these TFs orchestrate a shift in tumor cells from a state of rapid growth to a more invasive and metabolically adapted state. This change also reshapes the tumor’s surroundings, creating an environment that suppresses the immune system, largely by recruiting and activating specific immune cells called SPP1^+^ macrophages. This coordinated action between tumor cells and immune cells promotes cancer growth and immune evasion. Our findings highlight this TF–macrophage axis as a promising new target for developing more effective HCC therapies.

## 1. Introduction

Liver cancer caused approximately 870,000 new cases and 760,000 deaths worldwide in 2022 and ranked third in cancer mortality [1]. Hepatocellular carcinoma (HCC) accounts for ~90% of primary liver cancers [2]. Incidence is highest in East and Southeast Asia, where chronic hepatitis B remains prevalent [3]. Despite advances, HCC is often diagnosed late and recurs frequently; the global 5-year survival remains below 20% [3,4].

First-line therapy has shifted toward immune checkpoint inhibitor (ICI)–based combinations, particularly atezolizumab plus bevacizumab, which improves survival over prior standards [5,6,7]. However, objective response rates remain ~30%, and primary or acquired resistance is common. Heterogeneity in tumor-intrinsic programs and TME composition likely contributes to limited and transient responses [8].

Transcription factors (TFs) orchestrate gene-expression programs governing proliferation, survival, differentiation, and cell death [9]. Dysregulated TF activity underlies hallmark cancer behaviors, including unchecked proliferation, apoptosis resistance, invasion, metastasis, and angiogenesis [10]. Classical examples include loss of TP53 tumor suppression [11], MYC-driven metabolic amplification [12], and NF-κB–mediated inflammatory reprogramming [13,14].

Here, we integrated large public cohorts with single-cell genomics to define TF networks that drive malignant phenotypes in HCC. We focused on TF activity within malignant epithelial cells, mapped downstream regulon targets to functional pathways, and examined crosstalk with immune and stromal compartments. Our goal was to delineate tumor-intrinsic TF circuits, their spatial/temporal dynamics, and their impact on the TME to nominate actionable axes for therapy.

## 2. Materials and Methods

### 2.1. Study Cohort and Ethical Approval

We retrieved single-cell and spatial transcriptomic data from HCCDB v2.0 (lifeome.net), which aggregates bulk, single-cell, and spatial datasets with curated metadata. HCCDB v2.0 archives 182,832 single cells across three scRNA-seq datasets and 69,352 Visium spots from 17 tissue sections in 5 patients [15]. In addition, bulk RNA-seq data were obtained from the TCGA-LIHC cohort via the public XENA platform (xena.ucsc.edu), and the HCCDB25 dataset was used as an external validation cohort. Data are de-identified and publicly available; therefore, no additional IRB approval was required.

### 2.2. Single-Cell RNA-Seq Preprocessing and Quality Control

We used curated, QC-filtered matrices from HCCDB v2.0 for downstream analyses. HCCDB quality control removed cells with mitochondrial RNA fraction >20% and extreme feature counts (<200 or >10,000), followed by batch correction (Harmony) and Seurat-based clustering, yielding 182,832 cells after QC [16]. For spatial data, HCCDB provides 69,352 normalized spots across 17 sections (4 normal-adjacent, 4 leading-edge, 4 tumor core, 1 portal vein tumor thrombus, 4 intact tumor nodules). We retained HCCDB annotations and QC masks and applied subclustering, regulon inference, GSVA, pseudotime, and ligand–receptor analyses on these matrices.

### 2.3. Calling Malignant Epithelium by Copy-Number Aberrations

Within epithelial cells, malignant versus normal labels were assigned using CopyKAT v1.1.0 (default parameters). Aneuploid cells were labeled malignant; diploid cells were designated hepatocytes or cholangiocytes by canonical markers. CNV calls were cross-checked with inferCNV v1.24.0 (cutoff 0.1, denoise TRUE). 

### 2.4. Differential Expression and Gene-Set Analyses

Differentially expressed genes were identified with Seurat::FindMarkers (Wilcoxon; min.pct = 0.30; lock threshold = 1). P values were adjusted by the Benjamini–Hochberg method (FDR < 0.05). Gene-set variation analysis (GSVA v2.2.0) used msigdbr (v25.1.1). TF–Hallmark mappings were generated by intersecting regulon target sets with MSigDB Hallmarks and by GSVA/over-representation tests (Fisher’s exact; FDR < 0.05). Visualizations used tidygraph v1.3.1. In addition, comparisons of TF expression between normal and tumor samples in TCGA were performed using GEPIA (http://gepia.cancer-pku.cn/) [17].

### 2.5. Transcription-Factor Regulon Inference (SCENIC)

Regulatory networks were inferred with SCENIC/pySCENIC (v0.12.1; Python 3.9) [18]. GRNBoost2 was used for network learning, motif enrichment was assessed against RcisTarget (hg38-500bp and hg38-10kb), and regulon activity was scored by AUCell. For each epithelial subtype (hepatocyte, cholangiocyte, malignant), the top 50 TFs by regulon specificity score (RSS) were retained.

### 2.6. Stemness, Cell-Cycle State, and Functional Scoring

Differentiation potential was quantified with CytoTRACE2 (v1.1.0). Cell-cycle phase was assigned by Seurat:CellCycleScoring using canonical S and G2/M gene lists; proportions were compared by χ^2^ or Fisher’s exact tests. Functional programs (EMT, hypoxia, glycolysis, TNFα–NF-κB, etc.) were quantified by GSVA module scores.

### 2.7. Pseudotime Trajectory Analysis

Trajectories were reconstructed with Monocle2 v2.36.0 using ordering genes differentially expressed across malignant clusters (q < 1 × 10^−3^). CD4^+^ T-cell trajectories were inferred with Monocle3 (v1.4.26). Monocyte/macrophage trajectories were built within Monocle3 after extracting myeloid cells and applying learn_graph; cells were ordered by shortest paths through the principal graph. CD4^+^ naive T cells (TN) and CD14^+^ monocytes were used as roots.

### 2.8. Cell-Cell Interactions Analysis

Cell-cell interactions were inferred with CellChat (v2.1.2) using curated ligand–receptor pairs [19,20]. Overexpressed ligands/receptors were mapped to a PPI network to identify significant interactions via probabilistic modeling and permutation testing. Bubble plots were used for visualization. netAnalysis_contribution quantified LR pair contributions, and netVisual_circle traced the origin and targets of ligand signals.

## 3. Results

### 3.1. Single-Cell Characterization of the HCC Microenvironment

**Figure 1:**
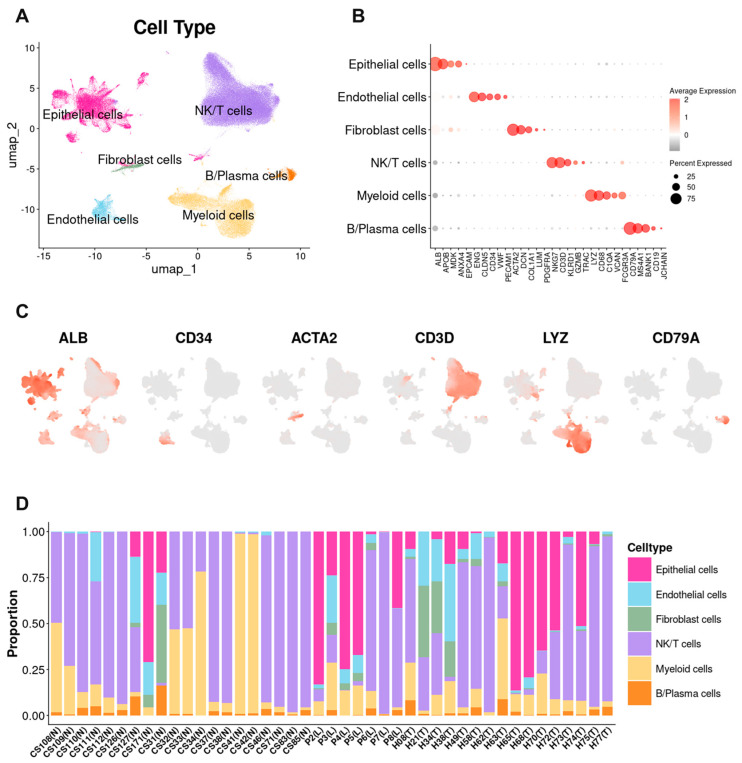
Single cell atlas of HCC and regional composition across normal-leading edge-tumor. (**A**) UMAP colored by major lineages. (**B**,**C**) Canonical marker expression validating lineages. (**D**) Patient-wise composition profiles showing consistent N-L-T trends. Abbreviations: N, normal; L, leading edge; T, tumor.

We profiled single-cell and spatial transcriptomes from normal (N), leading-edge (L), and tumor-core (T) regions (Figure S1A), resolving six major lineages by unsupervised embedding and canonical markers—epithelial, T/NK, myeloid, fibroblast, endothelial, and B/plasma cells (Figure 1A). Among 163,808 single cells, T/NK (~87,000) and myeloid (~30,500) populations comprised >70%; the epithelial compartment included 33,853 cells (~20%). Fibroblast and endothelial cells were less abundant, and B/plasma cells were infrequent (~3000) (Figure 1B,C). The epithelial fraction contained normal hepatocytes and cholangiocytes and malignant epithelial cells, which were further analyzed downstream. Across N → L → T, we observed consistent compositional shifts. Epithelial cells increased in T, reflecting tumor expansion. T/NK and myeloid fractions decreased from N to L/T, with a modest rebound from L to T, consistent with immune reorganization rather than uniform infiltration. Endothelial cells, fibroblasts, and B/plasma cells remained low overall but displayed focal accumulations in some patients. Patient-wise profiles confirmed these trends (Figure 1D).

### 3.2. Malignant Epithelium–Specific Transcription Factors and Prognostic Relevance

**Figure 2:**
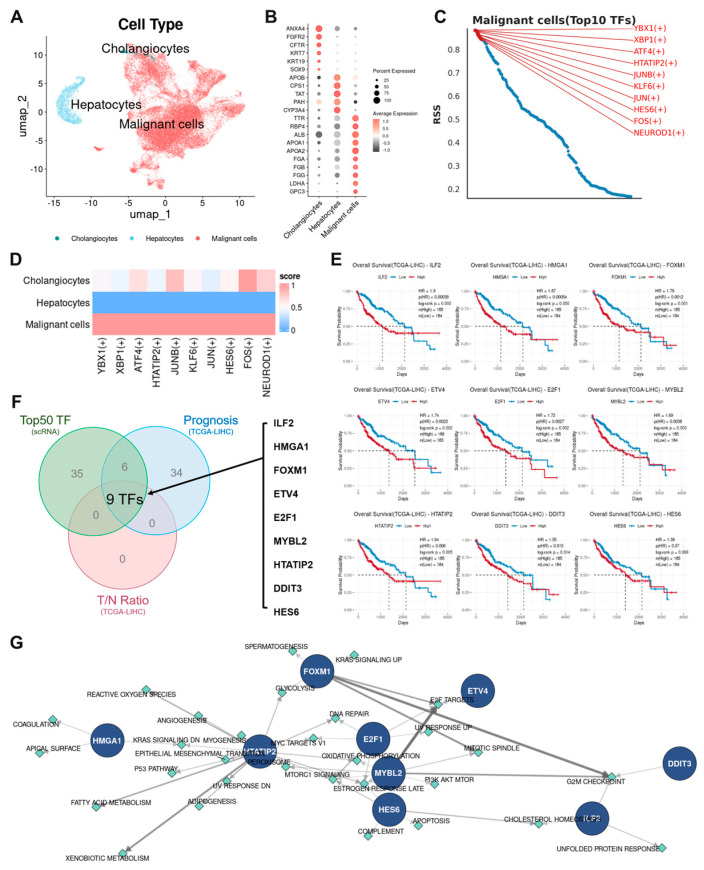
Identification of malignant epithelium–specific TFs and prognostic relevance. (**A**) Epithelial UMAP resolving hepatocytes, cholangiocytes, and malignant cells; CopyKAT CNV status (diploid vs. aneuploid) (see also Figure S1B). (**B**) Lineage markers supporting hepatocyte/cholangiocyte identities. (**C**) SCENIC regulon activity heatmap (top 10 TFs per epithelial subtype) (see also Figure S1C). (**D**) Minimal overlap among subtype-restricted TFs. (**E**) Kaplan–Meier overall survival. (**F**) Final nine TFs: ILF2, HMGA1, FOXM1, ETV4, E2F1, MYBL2, HTATIP2, DDIT3, HES6. (**G**) TF-Hallmark mapping network (see also Figure S1H).

We re-analyzed 33,853 epithelial cells. CopyKAT separated diploid normal lineages (hepatocytes, cholangiocytes) from aneuploid malignant epithelium (Figure 2A and Figure S1B), enabling classification into hepatocytes (2389), cholangiocytes (330), and malignant cells (31,134) (Figure 2A,B). SCENIC identified subtype-restricted regulons (top 50 TFs per subtype) (Figure 2C and Figure S1C; Table S1). Overlap across hepatocyte, cholangiocyte, and malignant TF lists was minimal (Figure 2D and Figure S1D), indicating a distinct malignant regulatory program. Clinical relevance in TCGA-LIHC revealed that higher expression of a subset of malignant-cell TF candidates associated with worse overall survival (Figure S1E). Tumor-versus-normal overexpression further refined a nine-TF panel—ILF2, HMGA1, FOXM1, ETV4, E2F1, MYBL2, HTATIP2, DDIT3, and HES6—consistently upregulated in tumors and associated with adverse prognosis (Figure S1F). In the TCGA-LIHC cohort, we confirmed that high-expression groups of all nine TFs were associated with poorer survival (Figure 2E,F). We further validated these unfavorable survival patterns in an independent cohort of 158 HCC patients from HCCDB25, where all nine TFs similarly showed significant associations with worse clinical outcomes (Figure S1G). Mapping of regulon targets to MSigDB Hallmarks showed FOXM1/E2F1/MYBL2 linking to proliferative circuits (G2/M, E2F targets, mitotic spindle), HMGA1/ETV4 aligning with EMT/hypoxia/TNF-α–NF-κB, and additional enrichments for glycolysis, apoptosis, and angiogenesis (Figure 2G and Figure S1H).

### 3.3. TF Activity–Defined Malignant Heterogeneity and Evolutionary Trajectories

**Figure 3:**
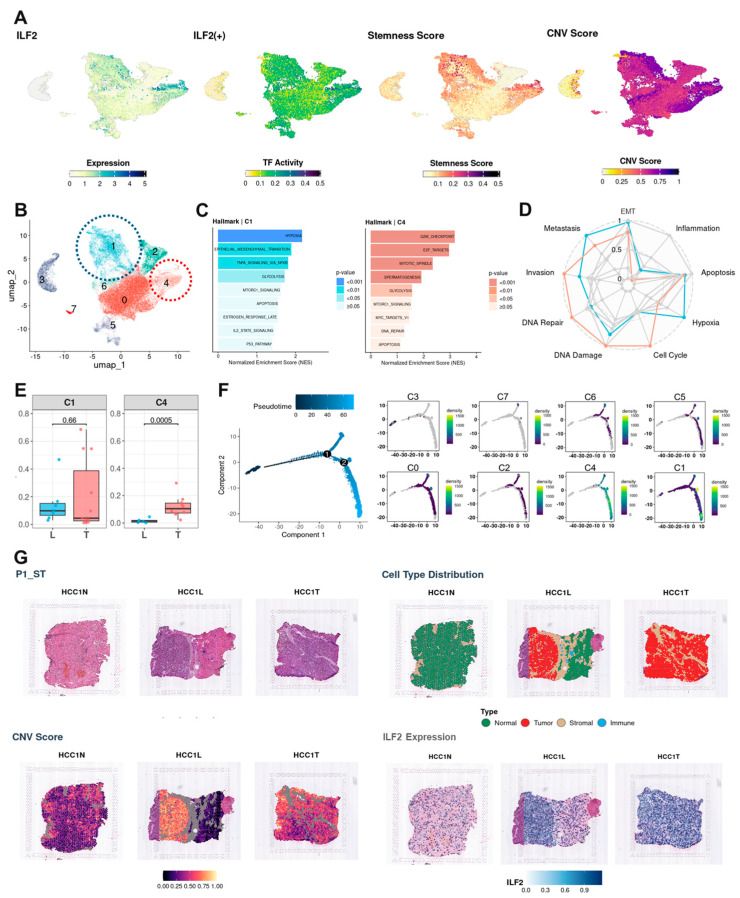
TF activity defined malignant states, functional programs, and spatial/temporal context. (**A**) UMAP overlays of regulon activity and mRNA expression for the nine TFs; elevated CNV and CytoTRACE stemness (see also Figure S2A). (**B**) Subclustering C0–C7; TF-high cells localize to C1 and C4. (**C**) Hallmarks: C1 (hypoxia, EMT, TNF-α/NF-κB, glycolysis) vs. C4 (G2/M, E2F targets, mitotic spindle). (**D**) CancerSEA functional scores. (**E**) Regional enrichment of C1/C4 (higher in T). (**F**) Pseudotime trajectory from C0 → C4 → C1; late pseudotime gene functions (see also Figure S2D). (**G**) Visium validation across four patients (see also Figure S3A,B). In panels (**B**) through (**D**), the blue color denotes Cluster 1, whereas the coral color represents Cluster 4. Abbreviations: L, leading edge; T, tumor.

UMAP overlays of regulon activity and mRNA expression highlighted a TF-high niche with elevated CNV scores and higher CytoTRACE-inferred stemness (Figure 3A and Figure S2A). Subclustering partitioned epithelial cells into eight groups (C0–C7), with TF-high cells concentrated in C1 (blue color cluster) and C4 (coral color cluster) (Figure 3B). Differential expression separated two dominant malignant phenotypes (Figure 3C and Figure S2B):

- C1 (invasive/metabolic/inflammatory): SPP1, TM4SF1, LGALS3, AGR2, G6PD, PKM, SLC2A1, NQO1, VEGFA, CAV1; enriched for hypoxia, EMT, TNF-α via NF-κB, glycolysis.
- C4 (proliferative): MKI67, TOP2A, BIRC5, CDC20, CDK1, CCNB1/2, PLK1, AURKA/B, TPX2, RRM2, MYBL2; enriched for G2/M checkpoint, E2F targets, mitotic spindle.

CancerSEA scores were concordant (C1 highest for EMT, metastasis, hypoxia; C4 highest for invasion, DNA damage/repair, cell cycle) (Figure 3D). Spatially, both C1 and C4 were significantly enriched in T relative to L (malignant cells were rare in N), indicating preferential accumulation of TF-high malignant states in tumor cores (Figure 3E). Cell-cycle assignment further separated the states (C4 enriched for G2/M; C1 enriched for G0/G1) (Figure S2C). Monocle2 pseudotime suggested a trajectory from C0 (hepatocytes) → C4 (proliferative) → C1 (invasive/EMT-like) with late-pseudotime genes enriched for cell-cycle checkpoints, mesenchymal differentiation, and extracellular matrix organization (Figure 3F and Figure S2D). Visium data across four patients validated higher expression of all nine TFs in tumor-annotated spots (high CNV) relative to normal-containing regions (Figure 3G and Figure S3A,B).

### 3.4. Regional Distribution of T/NK Cell Subtypes and Functional Reprogramming of CD4^+^ T Cells in the Tumor Microenvironment

**Figure 4:**
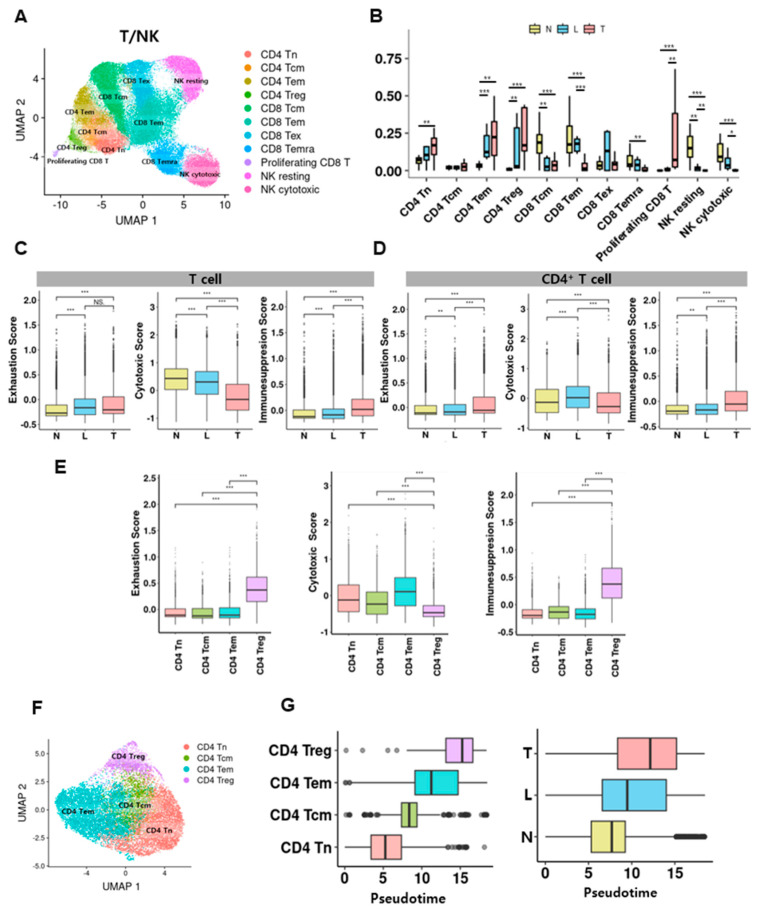
Functional states and pseudotime of intra-tumoral T and CD4^+^ T-cell subsets. (**A**) UMAP of T/NK phenotypes. (**B**) Regional abundance. Statistical significance was indicated as follows: * *p* < 0.05, ** *p* < 0.01, *** *p* < 0.001 (two-sided Wilcoxon rank-sum test). Non-significant comparisons were not displayed. (**C**) Exhaustion, cytotoxicity, and immunosuppresion scores across positions. Statistical significance was indicated as follows: ** *p* < 0.01, *** *p* < 0.001 (two-sided Wilcoxon rank-sum test). (**D**) Same for CD4^+^ T cells. Statistical significance was indicated as follows: *** *p* < 0.001 (two-sided Wilcoxon rank-sum test). (**E**) Scores across CD4^+^ subsets. (**F**) UMAP of CD4^+^ subsets. (**G**) Pseudotime distributions by subset and region.

Subclustering of 69,636 T/NK cells identified CD4^+^, CD8^+^, and NK subsets with canonical markers: FOXP3/IL2RA for CD4 Treg; PDCD1/LAG3 for CD8 Tex; GNLY/NKG7 for NK (Supplementary Figure S4A). UMAP revealed CD4 TN, TCM, TEM, Treg; CD8 TEM, TEX, TEMRA; proliferating CD8; resting and cytotoxic NK (Figure 4A). CD4 TEM, CD4 Treg, and proliferating CD8 T cells were enriched in the T group (Figure 4B and Figure S4B,C). Functional scoring showed increased exhaustion and immunosuppression and reduced cytotoxicity in T cells and CD4+ T cells, within T regions (Figure 4C,D). Among CD4^+^ T cell subsets, Treg exhibited the highest exhaustion/immunosuppression and the lowest cytotoxicity, whereas TEM displayed the highest cytotoxicity (Figure 4E). Monocle3 trajectories initiated from CD4 TN indicated a path TN → TCM → TEM → Treg, with normal-region cells at early pseudotime and tumor-core cells at terminal states (Figure 4F,G and Figure S4D,E).

### 3.5. SPP1^+^ Macrophages Represent a Dominant Immunosuppressive Subset Within the Tumor Core

**Figure 5:**
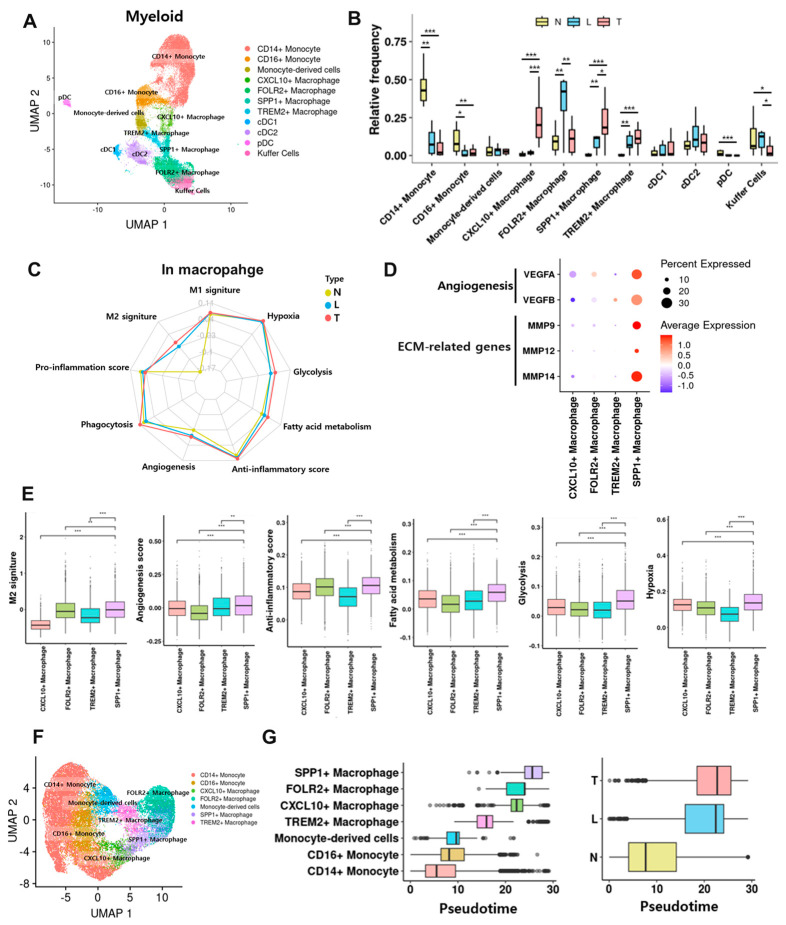
Functional states and pseudotime of intra-tumoral myeloid subsets. (**A**) UMAP of myeloid phenotypes. (**B**) Regional abundance. Statistical significance was indicated as follows: * *p* < 0.05, ** *p* < 0.01, *** *p* < 0.001 (two-sided Wilcoxon rank-sum test). Non-significant comparisons were not displayed. (**C**) Macrophage process signatures by region. (**D**) Angiogenesis/ECM gene expression across macrophage subsets. (**E**) M2, angiogenesis, anti-inflammatory, fatty acid metabolism, glycolysis, and hypoxia scores across subsets. Statistical significance was indicated as follows: ** *p* < 0.01, *** *p* < 0.001 (two-sided Wilcoxon rank-sum test). (**F**) UMAP of monocyte/macrophage phenotypes. (**G**) Pseudotime distributions by subset and region.

Clustering of 29,594 myeloid cells delineated CD14^+^/CD16^+^ monocytes, macrophage subtypes (CXCL10^+^, FOLR2^+^, TREM2^+^, SPP1^+^), and dendritic cells (Figure 5A and Figure S5A). SPP1^+^, CXCL10^+^, and TREM2^+^ macrophages were enriched in the T group, whereas CD14^+^/CD16^+^ monocytes and FOLR2^+^ macrophages were relatively abundant in N or L (Figure 5B and Figure S5B,C). Macrophages in the T group displayed higher M2, angiogenic, anti-inflammatory, hypoxic, glycolytic, and fatty acid metabolism signatures, with attenuated pro-inflammatory responses (Figure 5C). SPP1^+^ macrophages showed the strongest upregulation of angiogenesis (VEGFA/B) and ECM remodeling (MMP9/12/14) genes (Figure 5D) and the highest composite scores for M2, angiogenesis, anti-inflammatory, fatty acid metabolism, glycolysis, and hypoxia (Figure 5E). Monocle3 trajectories rooted in CD14^+^ monocytes suggested stepwise differentiation toward an SPP1^+^ terminal state—CD14^+^ → CD16^+^ → monocyte-derived macrophage → TREM2^+^ → CXCL10^+^ → FOLR2^+^ → SPP1^+^—with spatial transition from N to T group and progressive acquisition of immunosuppressive/angiogenic features (Figure 5F,G and Figure S5D,E).

### 3.6. Intercellular Crosstalk Between Tumor and Immune Cells Shapes a Distinct Invasive Tumor Microenvironment Through the SPP1 Signaling Pathway

**Figure 6:**
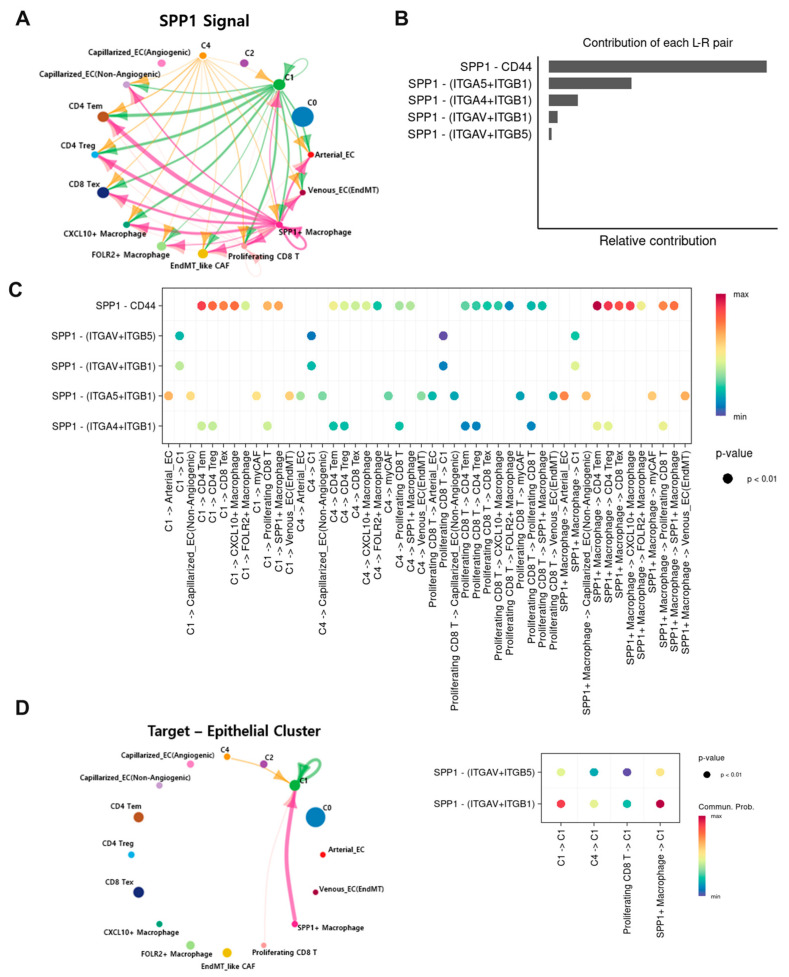
SPP1-mediated intercellular signaling in the TME. (**A**) Circos plot for the SPP1 pathway across cell types. (**B**) Ligand–receptor contribution to SPP1 signaling. (**C**) Dot plot of SPP1-mediated interactions via CD44 and integrins (ITGAV–ITGB5, ITGAV–ITGB1, ITGA5–ITGB1, ITGA4–ITGB1) between indicated cell types (*p* < 0.01). (**D**) Receiver-centric analysis specifying epithelial cells.

CellChat analysis across sixteen cell populations (C0/C1/C2/C4 epithelium; CD4 TEM, CD4 Treg, CD8 TEX; CXCL10^+^, FOLR2^+^, SPP1^+^ macrophages; arterial EC, venous EC [EndMT], capillarized EC [angiogenic and non-angiogenic]; myCAF) identified SPP1 signaling as a dominant axis. SPP1^+^ macrophages emerged as major sender cells; epithelial clusters C1 and C4 also contributed (Figure 6A and Figure S6). Signaling proceeded via CD44 and multiple integrins (ITGAV–ITGB1/ITGB5, ITGA5–ITGB1, ITGA4–ITGB1) toward epithelial, endothelial, CAF, T-cell, and macrophage recipients (Figure 6B,C). SPP1-CD44 interactions were prominent in exhausted CD8 and CD4 Treg recipients, suggesting contributions to T-cell dysfunction and Treg stabilization. Receiver-centric analysis for epithelial cells emphasized SPP1^+^ macrophage → epithelial (C1) signaling via ITGAV–ITGB1/ITGB5 (Figure 6D), consistent with integrin-dependent adhesion and motility.

## 4. Discussion

This study, through an integrated analysis of single-cell, spatial, and bulk transcriptomic data, has successfully identified a core network of nine tumor-specific transcription factors (TFs)—HTATIP2, HES6, ILF2, E2F1, MYBL2, DDIT3, FOXM1, HMGA1, and ETV4—that orchestrates the malignant programs of hepatocellular carcinoma (HCC). We have confirmed that this TF network is not merely a collection of individual genes but a sophisticated regulatory circuit that operates dynamically according to the stages of cancer progression. The primary significance of this research lies in its specific delineation of the tumor’s evolutionary trajectory, transitioning from an early proliferative state (cluster C4) to an invasive and metabolically adapted state (cluster C1).

The nine-TF panel we discovered is noteworthy as it comprises a combination of multi-dimensional regulators that comprehensively encompass the key hallmarks of cancer. FOXM1, E2F1, and MYBL2 are central factors in cell cycle regulation, and their activity directly explains the potent proliferative phenotype observed in the C4 cluster. This reconfirms their well-established roles at the single-cell level and demonstrates their cooperative action [21]. Concurrently, HMGA1 and ETV4 are deeply associated with epithelial–mesenchymal transition (EMT), hypoxia, and inflammatory signaling, acting as key drivers that confer the invasive characteristics of the C1 cluster [22]. The inclusion of a stress-response factor like DDIT3 suggests a mechanism by which the tumor adapts to and survives various internal and external stresses during its growth. Crucially, these transcriptomic states show strong concordance with the established histopathological framework of HCC. The invasive C1 state aligns with poorly differentiated, high-grade (Edmondson–Steiner grade III–IV) lesions, whereas the proliferative C4 state resembles well-differentiated tumors [23]. Moreover, the accumulation of SPP1^+^ macrophages in the C1 niche mirrors the immune-excluded, fibro-inflammatory features characteristic of aggressive histological subtypes [24,25], reinforcing the clinical relevance of our molecular classification.

The novelty of our study stems from revealing that these functionally heterogeneous TFs form a single, integrated network to systematically orchestrate a proliferation-to-invasion switch. This transition in the tumor-intrinsic program is accompanied by a dramatic remodeling of the tumor microenvironment (TME). Our research clearly demonstrates that tumor cells in the C1 state are not passive components of the TME but are active “architects” that construct an environment favorable for their own survival.

At the center of this process are SPP1^+^ macrophages. Our data suggest that C1 cells actively recruit SPP1^+^ macrophages into the tumor and polarize them toward an M2-like phenotype. These activated SPP1^+^ macrophages, in turn, neutralize T-cell function by secreting immunosuppressive cytokines and promoting the expansion of regulatory T cells (Tregs), while also facilitating tumor growth and metastasis by promoting angiogenesis and extracellular matrix (ECM) remodeling [26,27,28]. In particular, the SPP1-CD44 and SPP1-integrin interactions, identified through CellChat analysis, are the core molecular pathways mediating this complex intercellular communication. This provides a specific mechanism for how tumor cells and immune cells conspire to achieve both immune evasion and malignant progression.

While our multi-omics integration offers robust correlations, translating these insights into mechanistic evidence requires functional verification. Future studies will prioritize CRISPR/siRNA-mediated knockdown of core TFs (e.g., FOXM1, E2F1, HMGA1) in patient-derived organoids to confirm their role in driving the C4-to-C1 transition. Additionally, we plan to utilize co-culture systems to demonstrate that TF-high tumor cells actively polarize monocytes into SPP1^+^ macrophages and to validate that blocking the SPP1-CD44/integrin axis reverses immunosuppression, consistent with recent mechanistic studies [29,30]. These experimental efforts will be essential to bridge the gap between computational prediction and therapeutic application.

Nevertheless, the findings of this study offer significant implications for the development of therapeutic strategies for HCC. First, the 9-TF signature we have identified, or the ratio of C1/C4 clusters, has the potential to be utilized as a novel biomarker for predicting patient prognosis and therapeutic response. Second, the TF network itself or its downstream pathways could serve as direct therapeutic targets. While directly targeting TFs is technically challenging, the metabolic or signaling pathways they regulate (e.g., glycolysis) could be effective targets for existing or newly developed drugs. Finally, one of the most promising strategies is the blockade of SPP1-CD44 or SPP1-integrin interactions. Disrupting this key communication axis with antibodies or small-molecule compounds could dismantle the tumor’s immunosuppressive environment and maximize the efficacy of existing treatments like immune checkpoint inhibitors (ICIs), paving the way for novel combination therapy strategies. Future research should focus on evaluating the efficacy of these therapeutic strategies in preclinical models.

## 5. Conclusions

In this study, we have identified a core network of nine transcription factors that functions as a central engine for hepatocellular carcinoma progression. Our comprehensive analysis shows that these TFs collaboratively orchestrate a critical shift in cancer cell behavior, driving them from a proliferative state to a more aggressive, invasive phenotype. Crucially, our findings reveal that this internal tumor program actively reshapes the surrounding microenvironment. A key outcome of this remodeling is the recruitment and activation of SPP1^+^ macrophages, which establish an immunosuppressive shield that protects the tumor from immune attack and promotes the angiogenesis required for its sustained growth.

In conclusion, our work uncovers a powerful, interconnected axis between a tumor-intrinsic TF network and the co-opted immune cells in the TME. This provides a compelling model for how HCC establishes a permissive niche to thrive. The detailed molecular interactions we have pinpointed, particularly the SPP1-CD44 and SPP1-integrin pathways, represent tangible and promising targets for future therapeutic development. By disrupting this critical communication network, it may be possible to dismantle the tumor’s supportive environment, offering a novel strategy to overcome treatment resistance and improve clinical outcomes for patients with this challenging disease.

## Data Availability

All single-cell, spatial transcriptomic, and bulk RNA-seq datasets (including the HCCDB25 validation cohort) were obtained from the publicly accessible HCCDB v2.0 database (lifeome.net), which archives curated bulk, single-cell, and spatial transcriptomic datasets. These datasets are fully available for download by any user. The TCGA-LIHC bulk RNA-seq data were retrieved from the publicly available UCSC XENA platform (xena.ucsc.edu). All datasets used in this study are de-identified and openly accessible. No new data were generated.

## Supplementary Materials

The following supporting information can be downloaded at: https://www.mdpi.com/article/10.3390/cancers17233787/s1, Table S1: Target genes regulated by the nine tumor-restricted transcription factors (HTATIP2, HES6, ILF2, E2F1, MYBL2, DDIT3, FOXM1, HMGA1, and ETV4) identified by SCENIC analysis in malignant epithelial cells. Figure S1: Additional CNV and epithelial-subset validation; Figure S2: Extended TF activity maps, DEG details, and pseudotime gene functions; Figure S3: Spatial validation of tumor-specific TFs; Figure S4: Comprehensive characterization of T/NK cell states and CD4 T-cell pseudotime trajectories across regions; Figure S5: Comprehensive characterization of Myeloid cell states and pseudotime trajectories across regions; Figure S6: Fibroblast and endothelial subtyping and region-wise composition.
