# Supplementary material for: Transcription Factor Networks Drive Tumor Progression and Immune Microenvironment Remodeling in Hepatocellular Carcinoma
Source: Cancers (Basel). 2025 Nov 26;17(23):3787. doi: 10.3390/cancers17233787 (PMC12691271; doi:10.3390/cancers17233787)
Supplement: Supplementary file 1 [file cancers-17-03787-s001.zip › Supplementary_Figures.pdf]

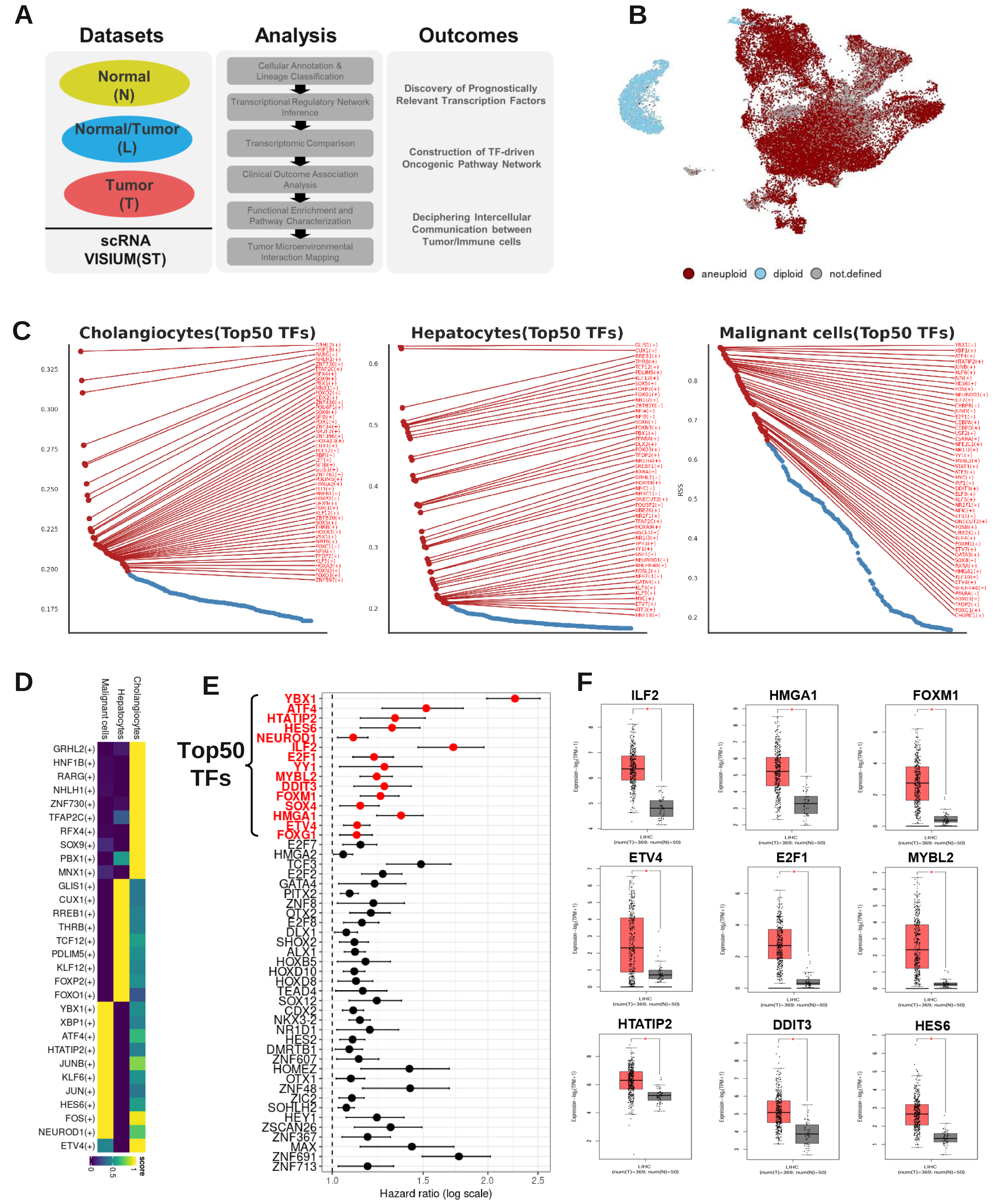
A
Datasets
Analysis
Outcomes
Normal (N)
Normal/Tumor (L)
Tumor (T)
scRNA VISIUM(ST)
Cellular Annotation & Lineage Classification
Transcriptional Regulatory Network Inference
Transcriptomic Comparison
Clinical Outcome Association Analysis
Functional Enrichment and Pathway Characterization
Tumor Microenvironmental Interaction Mapping
Discovery of Prognostically Relevant Transcription Factors
Construction of TF-driven Oncogenic Pathway Network
Deciphering Intercellular Communication between Tumor/Immune cells
B
aneuploid
diploid
not.defined
C
Cholangiocytes(Top50 TFs)
Hepatocytes(Top50 TFs)
Malignant cells(Top50 TFs)
RSS
D
Malignant cells
Hepatocytes
Cholangiocytes
GRHL2(+)
HNF1B(+)
RARG(+)
NHLH1(+)
ZNF730(+)
TFAP2C(+)
RFX4(+)
SOX9(+)
PBX1(+)
MNX1(+)
GLIS1(+)
CUX1(+)
RREB1(+)
THRB(+)
TCF12(+)
PDLIM5(+)
KLF12(+)
FOXP2(+)
FOXO1(+)
YBX1(+)
XBP1(+)
ATF4(+)
HTATIP2(+)
JUNB(+)
KLF6(+)
JUN(+)
HES6(+)
FOS(+)
NEUROD1(+)
ETV4(+)
score
E
Top50 TFs
YBX1
ATF4
HTATIP2
HES6
NEUROD1
ILF2
E2F1
YY1
MYBL2
DDIT3
FOXM1
SOX4
HMGA1
ETV4
FOXG1
E2F7
HMGA2
TCF3
E2F2
GATA4
PITX2
ZNF8
OTX2
E2F8
DLX1
SHOX2
ALX1
HOXB5
HOXD10
HOXD8
TEAD4
SOX12
CDX2
NKX3-2
NR1D1
HES2
DMRTB1
ZNF607
HOMEZ
OTX1
ZNF48
ZIC2
SOHLH2
HEY1
ZSCAN26
ZNF367
MAX
ZNF691
ZNF713
Hazard ratio (log scale)
F
ILF2
HMGA1
FOXM1
ETV4
E2F1
MYBL2
HTATIP2
DDIT3
HES6
LIHC

Figure S1

G

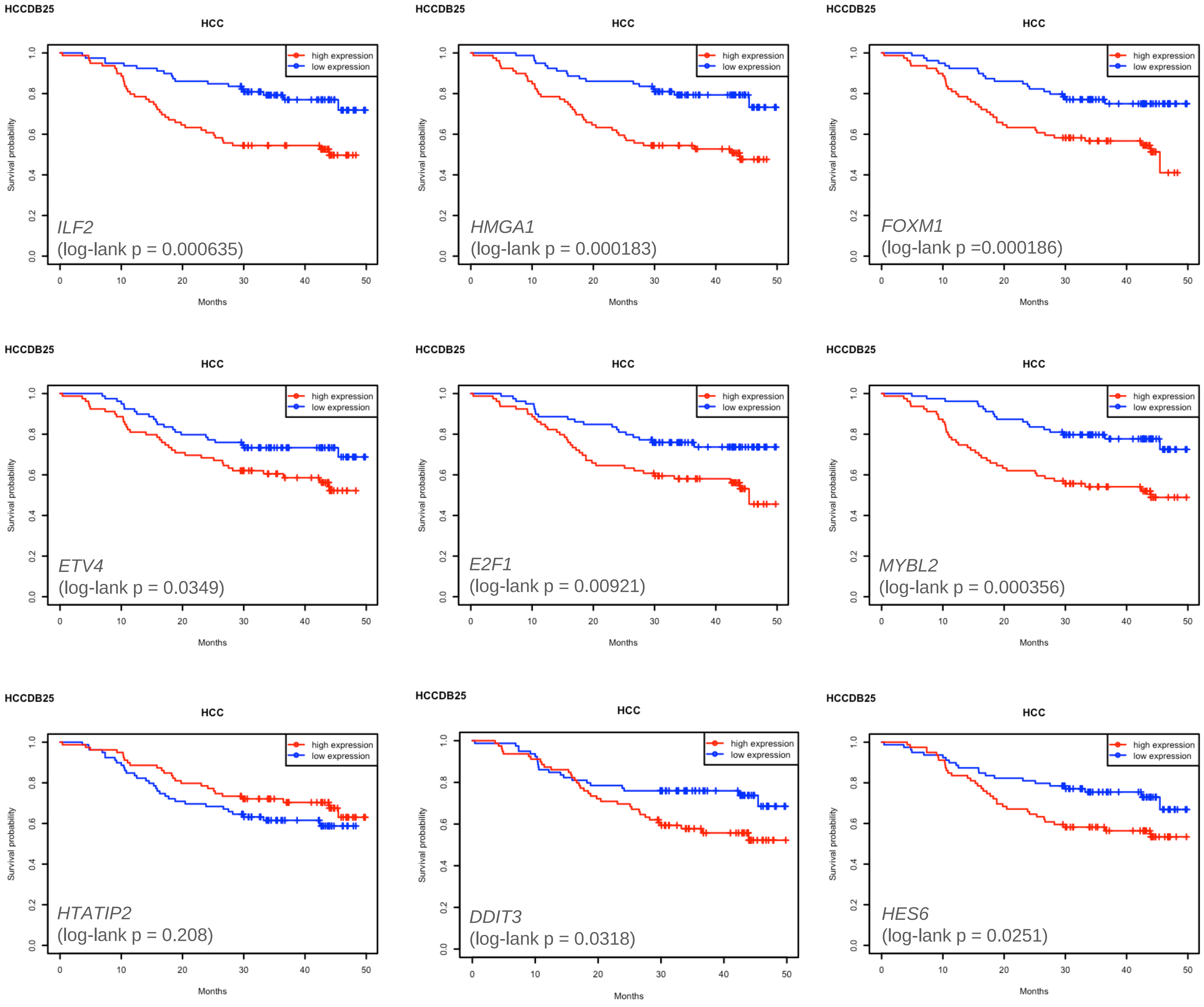

HCCDB25
HCC
high expression
low expression
Survival probability
Months
ILF2
(log-lank p = 0.000635)
HMGA1
(log-lank p = 0.000183)
FOXM1
(log-lank p =0.000186)
ETV4
(log-lank p = 0.0349)
E2F1
(log-lank p = 0.00921)
MYBL2
(log-lank p = 0.000356)
HTATIP2
(log-lank p = 0.208)
DDIT3
(log-lank p = 0.0318)
HES6
(log-lank p = 0.0251)

Figure S1

H

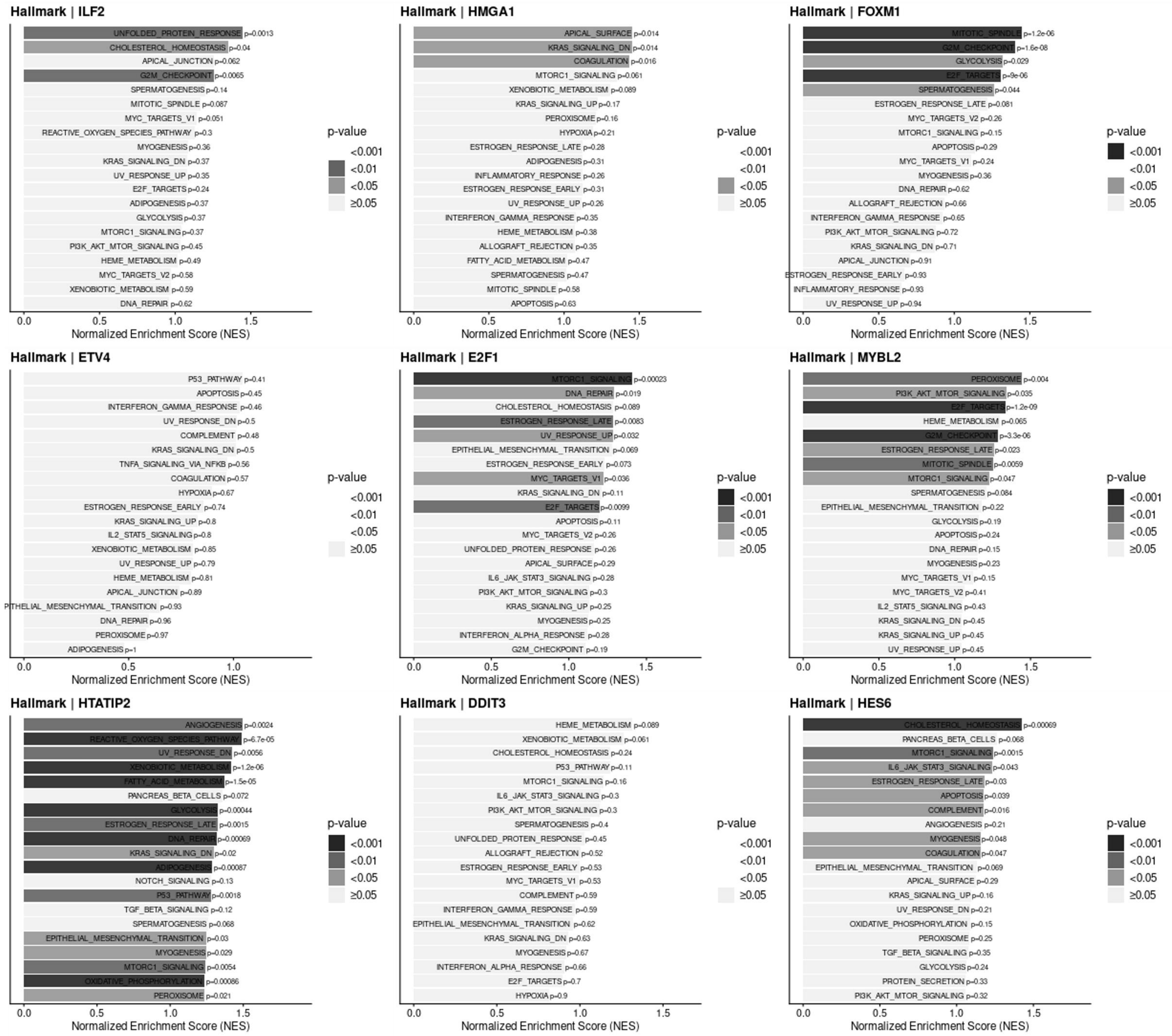
Hallmark | ILF2
UNFOLDED_PROTEIN_RESPONSE p=0.0013
CHOLESTEROL_HOMEOSTASIS p=0.04
APICAL_JUNCTION p=0.062
G2M_CHECKPOINT p=0.0065
SPERMATOGENESIS p=0.14
MITOTIC_SPINDLE p=0.087
MYC_TARGETS_V1 p=0.051
REACTIVE_OXYGEN_SPECIES_PATHWAY p=0.3
MYOGENESIS p=0.36
KRAS_SIGNALING_DN p=0.37
UV_RESPONSE_UP p=0.35
E2F_TARGETS p=0.24
ADIPOGENESIS p=0.37
GLYCOLYSIS p=0.37
MTORC1_SIGNALING p=0.37
PI3K_AKT_MTOR_SIGNALING p=0.45
HEME_METABOLISM p=0.49
MYC_TARGETS_V2 p=0.58
XENOBIOTIC_METABOLISM p=0.59
DNA_REPAIR p=0.62
Normalized Enrichment Score (NES)
p-value
<0.001
<0.01
<0.05
≥0.05
Hallmark | HMGA1
APICAL_SURFACE p=0.014
KRAS_SIGNALING_DN p=0.014
COAGULATION p=0.016
MTORC1_SIGNALING p=0.061
XENOBIOTIC_METABOLISM p=0.089
KRAS_SIGNALING_UP p=0.17
PEROXISOME p=0.16
HYPOXIA p=0.21
ESTROGEN_RESPONSE_LATE p=0.28
ADIPOGENESIS p=0.31
INFLAMMATORY_RESPONSE p=0.26
ESTROGEN_RESPONSE_EARLY p=0.31
UV_RESPONSE_UP p=0.26
INTERFERON_GAMMA_RESPONSE p=0.35
HEME_METABOLISM p=0.38
ALLOGRAFT_REJECTION p=0.35
FATTY_ACID_METABOLISM p=0.47
SPERMATOGENESIS p=0.47
MITOTIC_SPINDLE p=0.58
APOPTOSIS p=0.63
Hallmark | FOXM1
MITOTIC_SPINDLE p=1.2e-06
G2M_CHECKPOINT p=1.6e-08
GLYCOLYSIS p=0.029
E2F_TARGETS p=9e-06
SPERMATOGENESIS p=0.044
ESTROGEN_RESPONSE_LATE p=0.081
MYC_TARGETS_V2 p=0.26
MTORC1_SIGNALING p=0.15
APOPTOSIS p=0.29
MYC_TARGETS_V1 p=0.24
MYOGENESIS p=0.36
DNA_REPAIR p=0.62
ALLOGRAFT_REJECTION p=0.66
INTERFERON_GAMMA_RESPONSE p=0.65
PI3K_AKT_MTOR_SIGNALING p=0.72
KRAS_SIGNALING_DN p=0.71
APICAL_JUNCTION p=0.91
ESTROGEN_RESPONSE_EARLY p=0.93
INFLAMMATORY_RESPONSE p=0.93
UV_RESPONSE_UP p=0.94
Hallmark | ETV4
P53_PATHWAY p=0.41
APOPTOSIS p=0.45
INTERFERON_GAMMA_RESPONSE p=0.46
UV_RESPONSE_DN p=0.5
COMPLEMENT p=0.48
KRAS_SIGNALING_DN p=0.5
TNFA_SIGNALING_VIA_NFKB p=0.56
COAGULATION p=0.57
HYPOXIA p=0.67
ESTROGEN_RESPONSE_EARLY p=0.74
KRAS_SIGNALING_UP p=0.8
IL2_STAT5_SIGNALING p=0.8
XENOBIOTIC_METABOLISM p=0.85
UV_RESPONSE_UP p=0.79
HEME_METABOLISM p=0.81
APICAL_JUNCTION p=0.89
PITHELIAL_MESENCHYMAL_TRANSITION p=0.93
DNA_REPAIR p=0.96
PEROXISOME p=0.97
ADIPOGENESIS p=1
Hallmark | E2F1
MTORC1_SIGNALING p=0.00023
DNA_REPAIR p=0.019
CHOLESTEROL_HOMEOSTASIS p=0.089
ESTROGEN_RESPONSE_LATE p=0.0083
UV_RESPONSE_UP p=0.032
EPITHELIAL_MESENCHYMAL_TRANSITION p=0.069
ESTROGEN_RESPONSE_EARLY p=0.073
MYC_TARGETS_V1 p=0.036
KRAS_SIGNALING_DN p=0.11
E2F_TARGETS p=0.0099
APOPTOSIS p=0.11
MYC_TARGETS_V2 p=0.26
UNFOLDED_PROTEIN_RESPONSE p=0.26
APICAL_SURFACE p=0.29
IL6_JAK_STAT3_SIGNALING p=0.28
PI3K_AKT_MTOR_SIGNALING p=0.3
KRAS_SIGNALING_UP p=0.25
MYOGENESIS p=0.25
INTERFERON_ALPHA_RESPONSE p=0.28
G2M_CHECKPOINT p=0.19
Hallmark | MYBL2
PEROXISOME p=0.004
PI3K_AKT_MTOR_SIGNALING p=0.035
E2F_TARGETS p=1.2e-09
HEME_METABOLISM p=0.065
G2M_CHECKPOINT p=3.3e-06
ESTROGEN_RESPONSE_LATE p=0.023
MITOTIC_SPINDLE p=0.0059
MTORC1_SIGNALING p=0.047
SPERMATOGENESIS p=0.084
EPITHELIAL_MESENCHYMAL_TRANSITION p=0.22
GLYCOLYSIS p=0.19
APOPTOSIS p=0.24
DNA_REPAIR p=0.15
MYOGENESIS p=0.23
MYC_TARGETS_V1 p=0.15
MYC_TARGETS_V2 p=0.41
IL2_STAT5_SIGNALING p=0.43
KRAS_SIGNALING_DN p=0.45
KRAS_SIGNALING_UP p=0.45
UV_RESPONSE_UP p=0.45
Hallmark | HTATIP2
ANGIOGENESIS p=0.0024
REACTIVE_OXYGEN_SPECIES_PATHWAY p=6.7e-05
UV_RESPONSE_DN p=0.0056
XENOBIOTIC_METABOLISM p=1.2e-06
FATTY_ACID_METABOLISM p=1.5e-05
PANCREAS_BETA_CELLS p=0.072
GLYCOLYSIS p=0.00044
ESTROGEN_RESPONSE_LATE p=0.0015
DNA_REPAIR p=0.00069
KRAS_SIGNALING_DN p=0.02
ADIPOGENESIS p=0.00087
NOTCH_SIGNALING p=0.13
P53_PATHWAY p=0.0018
TGF_BETA_SIGNALING p=0.12
SPERMATOGENESIS p=0.068
EPITHELIAL_MESENCHYMAL_TRANSITION p=0.03
MYOGENESIS p=0.029
MTORC1_SIGNALING p=0.0054
OXIDATIVE_PHOSPHORYLATION p=0.00086
PEROXISOME p=0.021
Hallmark | DDIT3
HEME_METABOLISM p=0.089
XENOBIOTIC_METABOLISM p=0.061
CHOLESTEROL_HOMEOSTASIS p=0.24
P53_PATHWAY p=0.11
MTORC1_SIGNALING p=0.16
IL6_JAK_STAT3_SIGNALING p=0.3
PI3K_AKT_MTOR_SIGNALING p=0.3
SPERMATOGENESIS p=0.4
UNFOLDED_PROTEIN_RESPONSE p=0.45
ALLOGRAFT_REJECTION p=0.52
ESTROGEN_RESPONSE_EARLY p=0.53
MYC_TARGETS_V1 p=0.53
COMPLEMENT p=0.59
INTERFERON_GAMMA_RESPONSE p=0.59
EPITHELIAL_MESENCHYMAL_TRANSITION p=0.62
KRAS_SIGNALING_DN p=0.63
MYOGENESIS p=0.67
INTERFERON_ALPHA_RESPONSE p=0.66
E2F_TARGETS p=0.7
HYPOXIA p=0.9
Hallmark | HES6
CHOLESTEROL_HOMEOSTASIS p=0.00069
PANCREAS_BETA_CELLS p=0.068
MTORC1_SIGNALING p=0.0015
IL6_JAK_STAT3_SIGNALING p=0.043
ESTROGEN_RESPONSE_LATE p=0.03
APOPTOSIS p=0.039
COMPLEMENT p=0.016
ANGIOGENESIS p=0.21
MYOGENESIS p=0.048
COAGULATION p=0.047
EPITHELIAL_MESENCHYMAL_TRANSITION p=0.069
APICAL_SURFACE p=0.29
KRAS_SIGNALING_UP p=0.16
UV_RESPONSE_DN p=0.21
OXIDATIVE_PHOSPHORYLATION p=0.15
PEROXISOME p=0.25
TGF_BETA_SIGNALING p=0.35
GLYCOLYSIS p=0.24
PROTEIN_SECRETION p=0.33
PI3K_AKT_MTOR_SIGNALING p=0.32

Figure S1

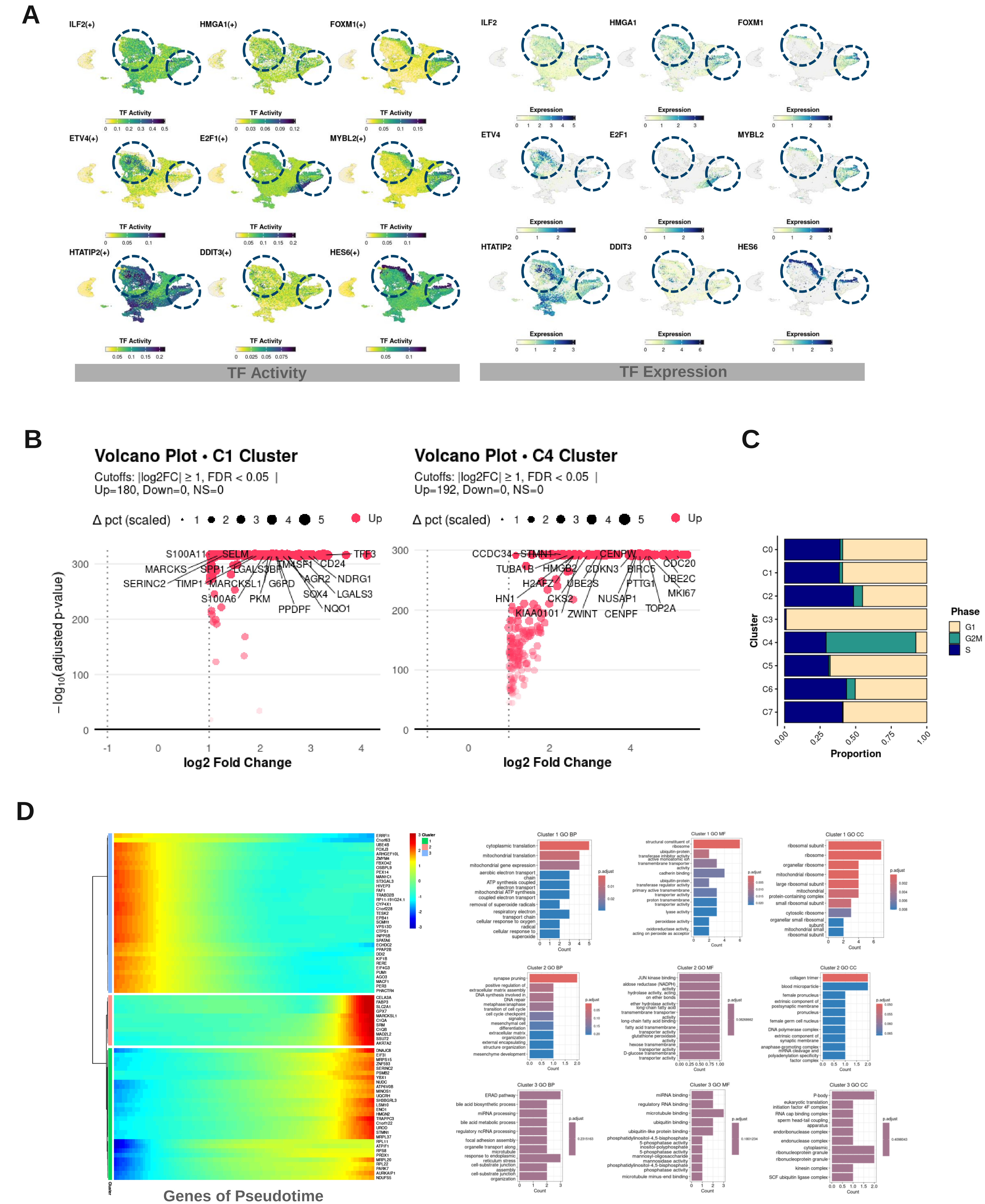
A
ILF2(+)
HMGA1(+)
FOXM1(+)
ETV4(+)
E2F1(+)
MYBL2(+)
HTATIP2(+)
DDIT3(+)
HES6(+)
TF Activity
ILF2
HMGA1
FOXM1
ETV4
E2F1
MYBL2
HTATIP2
DDIT3
HES6
Expression
TF Expression
B
Volcano Plot • C1 Cluster
Cutoffs: |log2FC| ≥ 1, FDR < 0.05 |
Up=180, Down=0, NS=0
Δ pct (scaled)
Up
−log10(adjusted p-value)
log2 Fold Change
Volcano Plot • C4 Cluster
Cutoffs: |log2FC| ≥ 1, FDR < 0.05 |
Up=192, Down=0, NS=0
C
Cluster
Proportion
Phase
G1
G2M
S
D
Genes of Pseudotime
Cluster 1 GO BP
Cluster 1 GO MF
Cluster 1 GO CC
Cluster 2 GO BP
Cluster 2 GO MF
Cluster 2 GO CC
Cluster 3 GO BP
Cluster 3 GO MF
Cluster 3 GO CC
p.adjust
Count

Figure S2

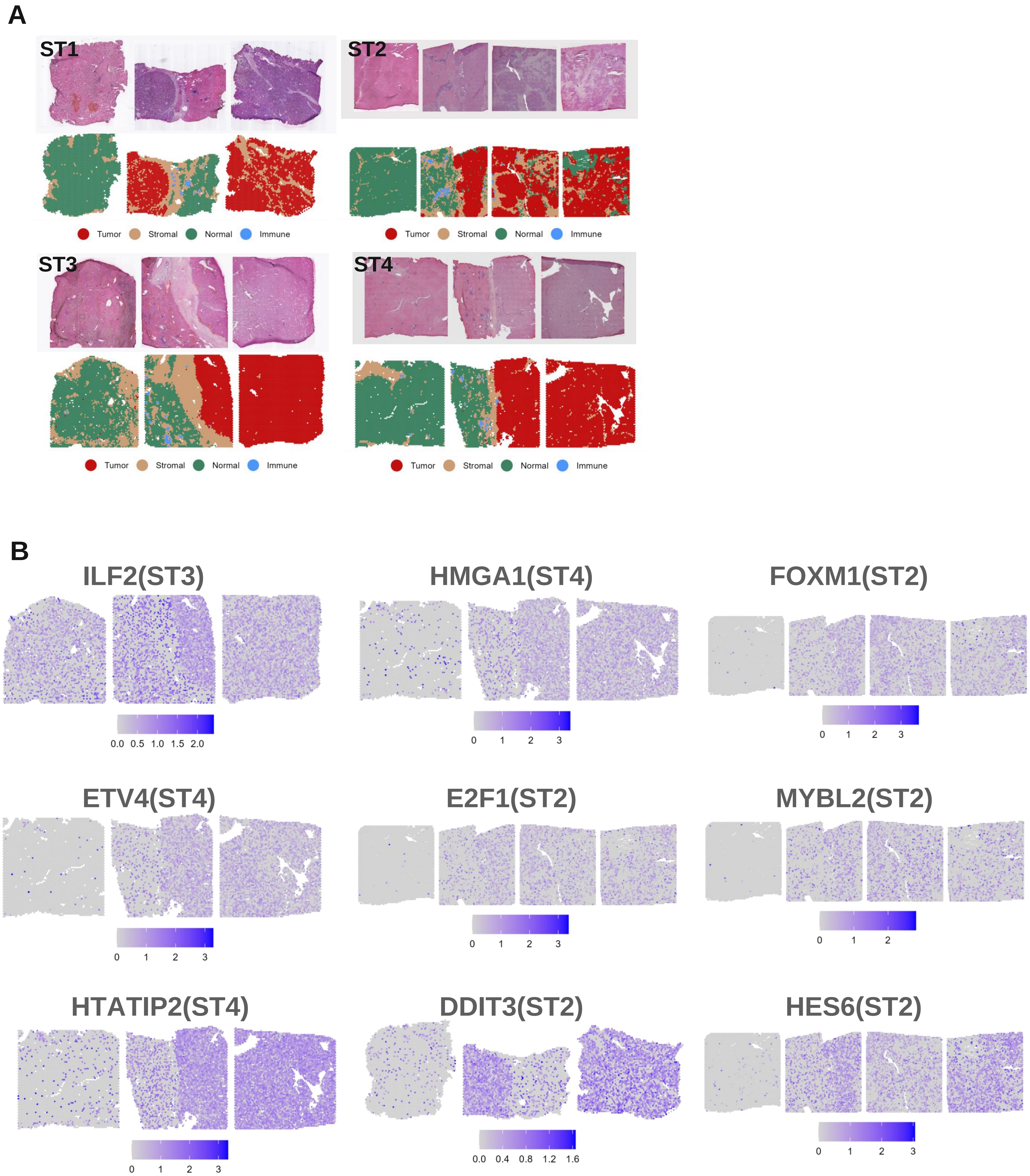
A
ST1
ST2
Tumor Stromal Normal Immune
Tumor Stromal Normal Immune
ST3
ST4
Tumor Stromal Normal Immune
Tumor Stromal Normal Immune
B
ILF2(ST3)
0.0 0.5 1.0 1.5 2.0
HMGA1(ST4)
0 1 2 3
FOXM1(ST2)
0 1 2 3
ETV4(ST4)
0 1 2 3
E2F1(ST2)
0 1 2 3
MYBL2(ST2)
0 1 2
HTATIP2(ST4)
0 1 2 3
DDIT3(ST2)
0.0 0.4 0.8 1.2 1.6
HES6(ST2)
0 1 2 3

Figure S3

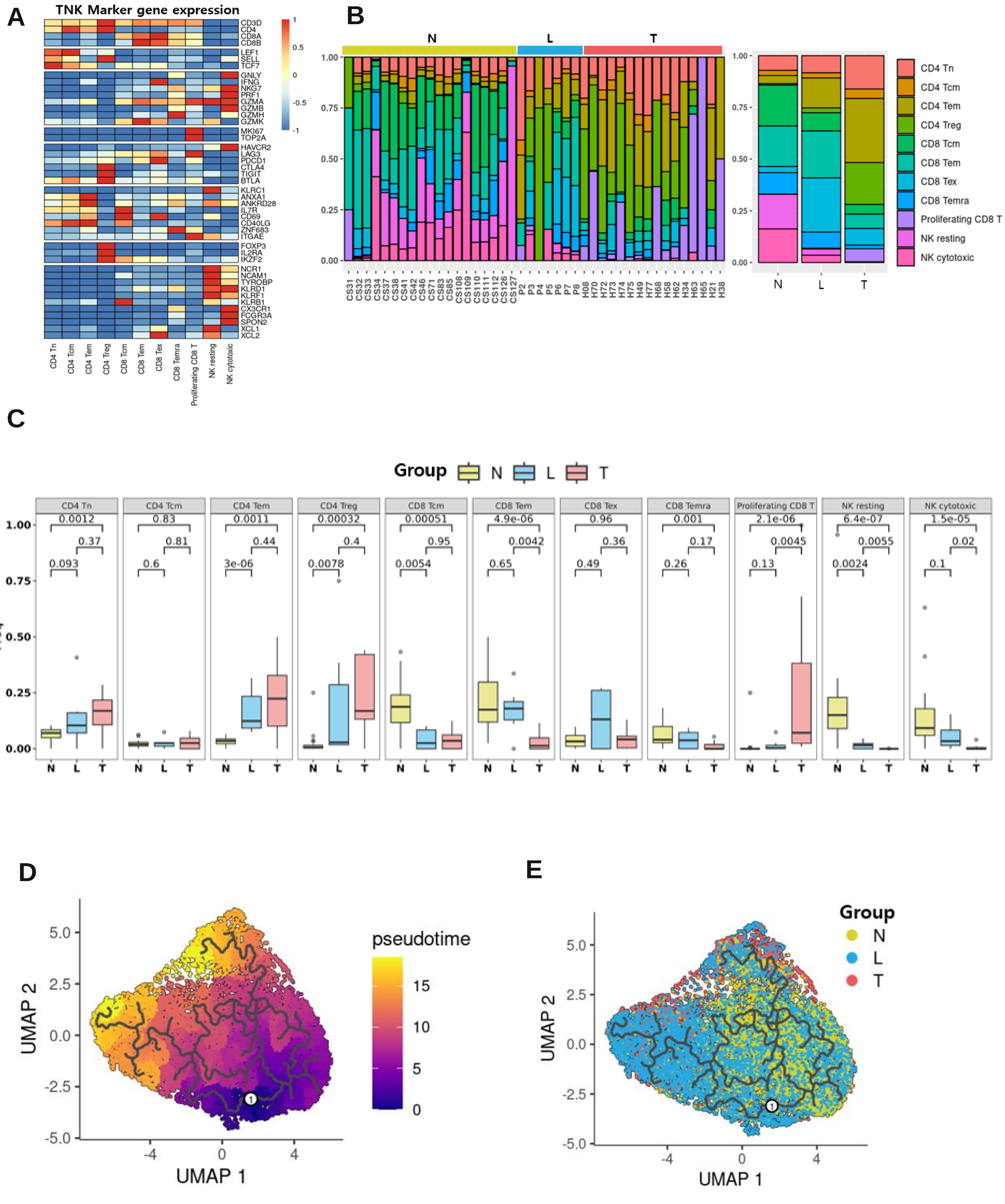
A
TNK Marker gene expression
CD3D
CD4
CD8A
CD8B
LEF1
SELL
TCF7
GNLY
IFNG
NKG7
PRF1
GZMA
GZMB
GZMH
GZMK
MKI67
TOP2A
HAVCR2
LAG3
PDCD1
CTLA4
TIGIT
BTLA
KLRC1
ANXA1
ANKRD28
IL7R
CD69
CD40LG
ZNF683
ITGAE
FOXP3
IL2RA
IKZF2
NCR1
NCAM1
TYROBP
KLRD1
KLRF1
KLRB1
CX3CR1
FCGR3A
SPON2
XCL1
XCL2
CD4 Tn
CD4 Tcm
CD4 Tem
CD4 Treg
CD8 Tcm
CD8 Tem
CD8 Tex
CD8 Temra
Proliferating CD8 T
NK resting
NK cytotoxic
B
N
L
T
CD4 Tn
CD4 Tcm
CD4 Tem
CD4 Treg
CD8 Tcm
CD8 Tem
CD8 Tex
CD8 Temra
Proliferating CD8 T
NK resting
NK cytotoxic
C
Group
N
L
T
Freq
D
pseudotime
UMAP 1
UMAP 2
E
Group
N
L
T
UMAP 1
UMAP 2

Figure S4

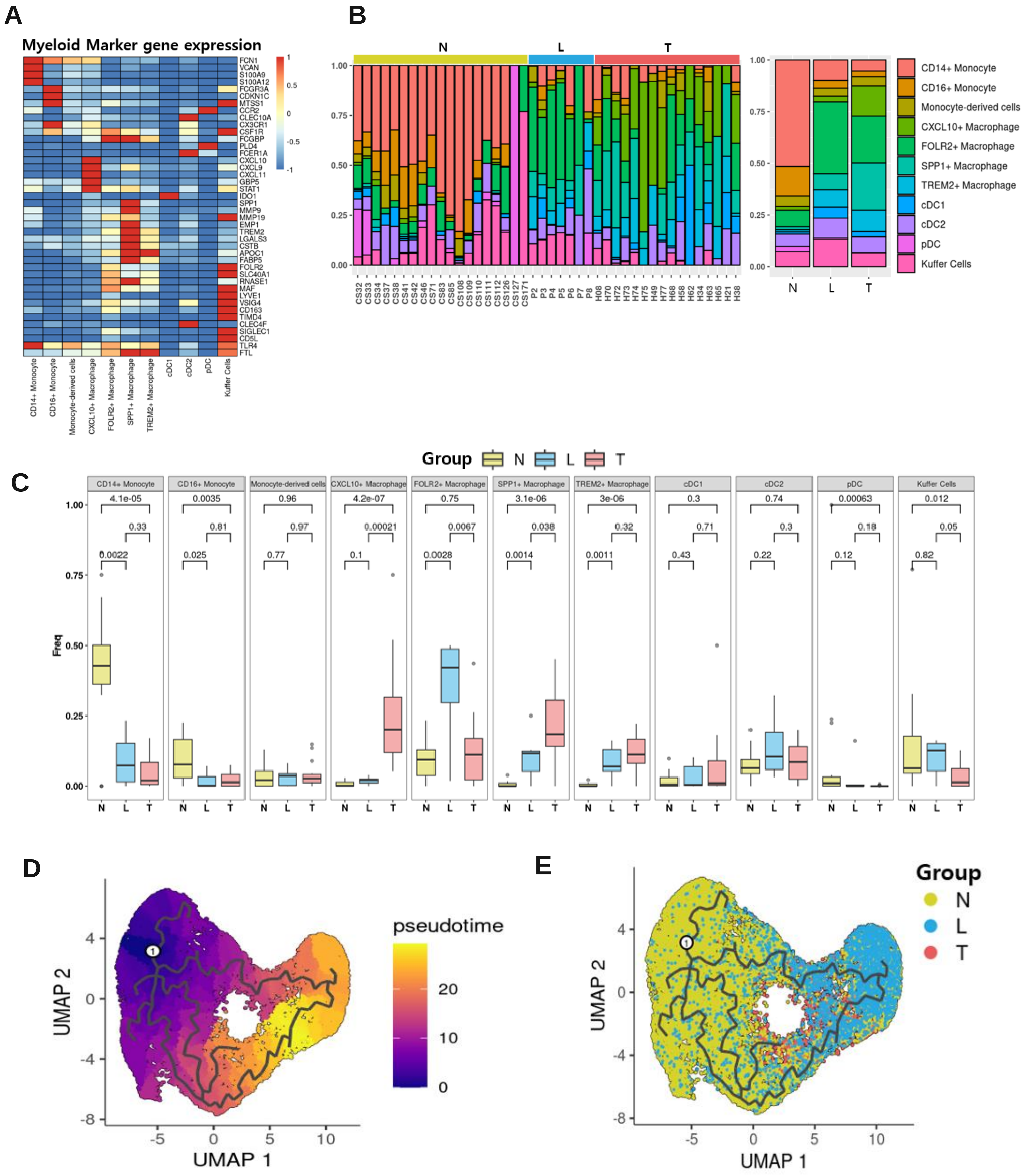

A
Myeloid Marker gene expression
FCN1
VCAN
S100A9
S100A12
FCGR3A
CDKN1C
MTSS1
CCR2
CLEC10A
CX3CR1
CSF1R
FCGBP
PLD4
FCER1A
CXCL10
CXCL9
CXCL11
GBP5
STAT1
IDO1
SPP1
MMP9
MMP19
EMP1
TREM2
LGALS3
CSTB
APOC1
FABP5
FOLR2
SLC40A1
RNASE1
MAF
LYVE1
VSIG4
CD163
TIMD4
CLEC4F
SIGLEC1
CD5L
TLR4
FTL
1
0.5
0
-0.5
-1
CD14+ Monocyte
CD16+ Monocyte
Monocyte-derived cells
CXCL10+ Macrophage
FOLR2+ Macrophage
SPP1+ Macrophage
TREM2+ Macrophage
cDC1
cDC2
pDC
Kuffer Cells
B
N
L
T
1.00
0.75
0.50
0.25
0.00
CS32 CS33 CS34 CS37 CS38 CS41 CS42 CS46 CS71 CS83 CS85 CS108 CS109 CS110 CS111 CS112 CS126 CS127 CS171 P2 P3 P4 P5 P6 P7 P8 H08 H70 H72 H73 H74 H75 H49 H77 H58 H68 H62 H34 H63 H65 H21 H38
CD14+ Monocyte
CD16+ Monocyte
Monocyte-derived cells
CXCL10+ Macrophage
FOLR2+ Macrophage
SPP1+ Macrophage
TREM2+ Macrophage
cDC1
cDC2
pDC
Kuffer Cells
C
Group N L T
CD14+ Monocyte
CD16+ Monocyte
Monocyte-derived cells
CXCL10+ Macrophage
FOLR2+ Macrophage
SPP1+ Macrophage
TREM2+ Macrophage
cDC1
cDC2
pDC
Kuffer Cells
4.1e-05 0.33 0.0022
0.0035 0.81 0.025
0.96 0.97 0.77
4.2e-07 0.00021 0.1
0.75 0.0067 0.0028
3.1e-06 0.038 0.0014
3e-06 0.32 0.0011
0.3 0.71 0.43
0.74 0.3 0.22
0.00063 0.18 0.12
0.012 0.05 0.82
Freq
1.00
0.75
0.50
0.25
0.00
N L T
D
pseudotime
20
10
0
UMAP 2
UMAP 1
4
0
-4
-8
-5
0
5
10
E
Group
N
L
T
UMAP 2
UMAP 1

Figure S5

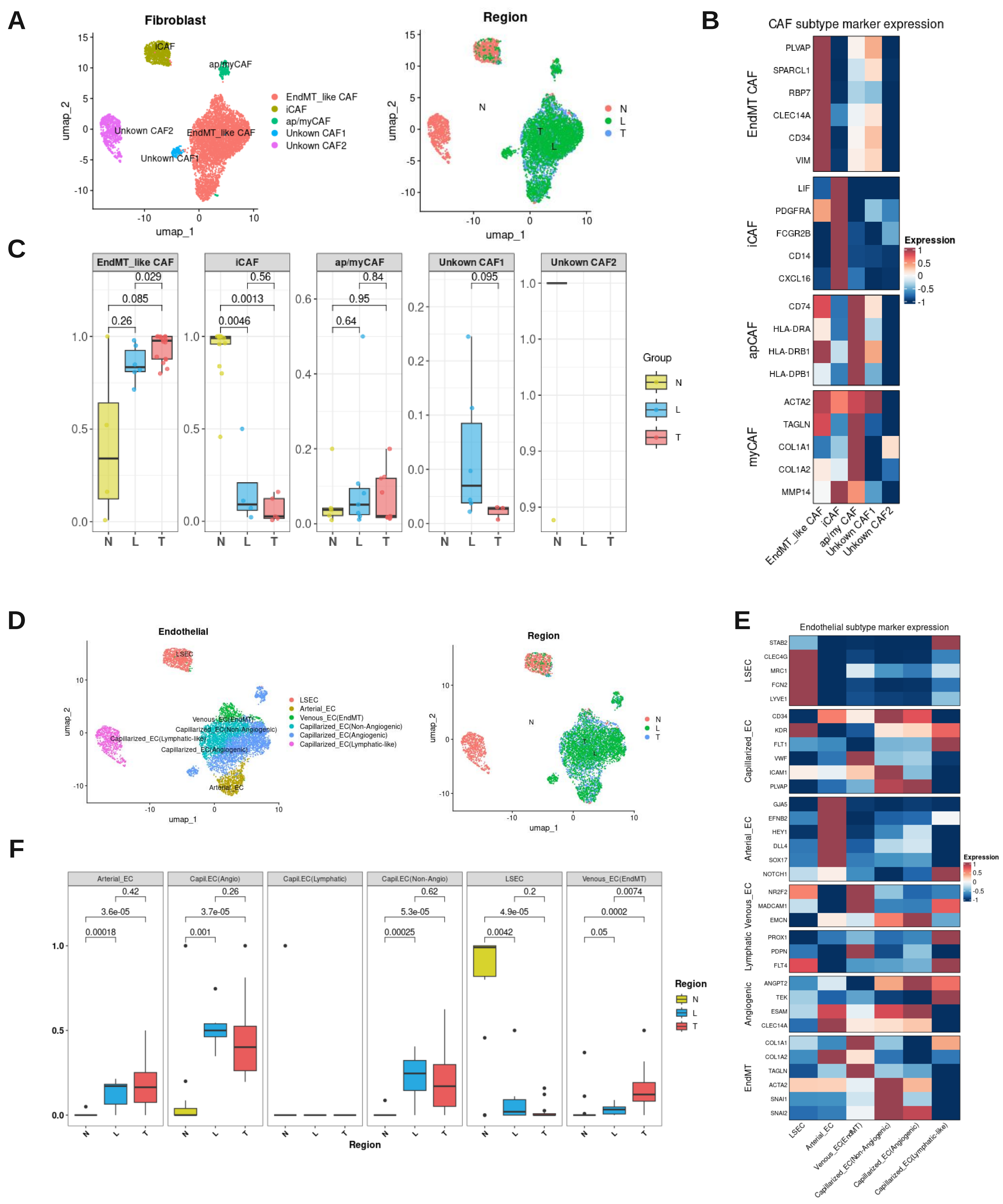
A
Fibroblast
iCAF
ap/myCAF
Unkown CAF2
EndMT_like CAF
Unkown CAF1
EndMT_like CAF
iCAF
ap/myCAF
Unkown CAF1
Unkown CAF2
umap_1
umap_2
Region
N
T
L
N
L
T
B
CAF subtype marker expression
EndMT CAF
PLVAP
SPARCL1
RBP7
CLEC14A
CD34
VIM
iCAF
LIF
PDGFRA
FCGR2B
CD14
CXCL16
apCAF
CD74
HLA-DRA
HLA-DRB1
HLA-DPB1
myCAF
ACTA2
TAGLN
COL1A1
COL1A2
MMP14
Expression
EndMT_like CAF
iCAF
ap/my CAF
Unkown CAF1
Unkown CAF2
C
EndMT_like CAF
iCAF
ap/myCAF
Unkown CAF1
Unkown CAF2
0.029
0.085
0.26
0.56
0.0013
0.0046
0.84
0.95
0.64
0.095
Group
N
L
T
D
Endothelial
LSEC
Arterial_EC
Venous_EC(EndMT)
Capillarized_EC(Non-Angiogenic)
Capillarized_EC(Angiogenic)
Capillarized_EC(Lymphatic-like)
umap_1
umap_2
Region
E
Endothelial subtype marker expression
LSEC
STAB2
CLEC4G
MRC1
FCN2
LYVE1
Capillarized_EC
CD34
KDR
FLT1
VWF
ICAM1
PLVAP
Arterial_EC
GJA5
EFNB2
HEY1
DLL4
SOX17
NOTCH1
Venous_EC
NR2F2
MADCAM1
EMCN
Lymphatic
PROX1
PDPN
FLT4
Angiogenic
ANGPT2
TEK
ESAM
CLEC14A
EndMT
COL1A1
COL1A2
TAGLN
ACTA2
SNAI1
SNAI2
Expression
F
Arterial_EC
Capil.EC(Angio)
Capil.EC(Lymphatic)
Capil.EC(Non-Angio)
LSEC
Venous_EC(EndMT)
0.42
3.6e-05
0.00018
0.26
3.7e-05
0.001
0.62
5.3e-05
0.00025
0.2
4.9e-05
0.0042
0.0074
0.0002
0.05
Region
N
L
T

Figure S6
