# Supplementary material for: Transcription Factor Networks Drive Tumor Progression and Immune Microenvironment Remodeling in Hepatocellular Carcinoma
Source: Cancers (Basel). 2025 Nov 26;17(23):3787. doi: 10.3390/cancers17233787 (PMC12691271; doi:10.3390/cancers17233787)
Supplement: Supplementary file 1 [file cancers-17-03787-s001.zip › Supplementary_Legend.pdf]

Legend of Supplementary Figure

Supplementary Figure S1. Additional CNV and epithelial-subset validation

(A) Schematic of sampling (normal, N; leading edge, L; tumor core, T) and analysis workflow. (B) CopyKAT CNV dotplot distinguishing diploid hepatocytes/cholangiocytes from aneuploid malignant epithelium. (C) Extended SCENIC regulon summaries supporting subtype-restricted TF activation. (D) SCENIC regulon activity heatmap (top 10 TFs per epithelial subtype). (E) Univariable Cox models in TCGA-LIHC for malignant-cell TF candidates (hazard ratios, 95% CI). (F) Tumor-versus-normal differential expression in TCGA-LIHC (Gepia2). (G) Kaplan–Meier overall survival curves for the nine TFs (log-rank) in validation set. (H) TF–Hallmark mapping of regulon targets. Proliferation circuits (G2/M checkpoint, E2F targets, mitotic spindle) predominate for FOXM1/E2F1/MYBL2; HMGA1/ETV4 align with EMT, hypoxia, and TNF-α/NF-κB; additional enrichments include glycolysis, apoptosis, and angiogenesis.

Supplementary Figure S2. Extended TF activity maps, DEG details, and pseudotime gene functions

(A) Per-TF regulon activity and expression overlays on UMAP, complementing Fig. 3A. (B) Expanded DEG heatmaps/dot plots for C1 (SPP1, LGALS3, AGR2, G6PD, PKM, SLC2A1, NQO1, VEGFA, CAV1, TM4SF1) and C4 (MKI67, TOP2A, BIRC5, CDC20, CDK1, CCNB1/2, PLK1, AURKA/B, TPX2, RRM2, MYBL2). (C) Cell-cycle phase distribution (C4 enriched for G2/M; C1 enriched for G0/G1). (D) GO/Hallmark enrichments among late-pseudotime genes (cell-cycle checkpoints, mesenchymal differentiation, ECM organization), supporting Fig. 3G.

Supplementary Figure S3. Spatial validation of tumor-specific TFs

(A) Visium spot classification (Tumor/Stromal/Immune/Normal) and CNV-inferred tumor areas. (B) Spatial expression maps and patient-level summaries demonstrating higher expression of ILF2, HMGA1, FOXM1, ETV4, E2F1, MYBL2, DDIT3, HES6, and HTATIP2 in tumor regions relative to normal-containing areas across all four patients.

Supplementary Figure S4. Comprehensive characterization of T/NK cell states and CD4 T-cell pseudotime trajectories across regions.

(A) Heatmaps showing expression of T/NK marker genes. (B) Relative abundance of T/NK subtype stratified by individual samples (left) different resions (right). (C) Boxplots showing the relative abundance of T/NK-cell phenotypes across different regions. Statistical significance was evaluated using a two-sided Wilcoxon rank-sum test. Only significant p-values are shown (*p < 0.05, **p < 0.01, ***p < 0.001). (D) UMAP visualization of $CD4^{+}$ T cells colored by pseudotime values, indicating a gradual state transition across the inferred trajectory. (E) UMAP visualization of different regions showing distinct spatial distributions along virtual time trajectories within CD4 T cells.

Supplementary Figure S5. Comprehensive characterization of Myeloid cell states and pseudotime trajectories across regions.

(A) Heatmaps showing expression of Myeloid marker genes. (B) Relative abundance of myeloid cell subtype stratified by individual samples (left) different resions (right). (C) Boxplots showing the relative abundance of myeloid cell subtypes across different regions. Statistical significance was evaluated using a two-sided Wilcoxon rank-sum test. Only significant p-values are shown (*p < 0.05, **p < 0.01, ***p < 0.001). (D) UMAP visualization of monocyte and macrophage colored by pseudotime values, indicating a gradual state transition across the inferred trajectory. (E) UMAP visualization of different regions showing distinct spatial distributions along virtual time trajectories within monocyte and macrophage.

Supplementary Figure S6. Fibroblast and endothelial subtyping and region-wise composition

Subclustering resolved discrete fibroblast states—portal-like, inflammatory/chemokine-rich, ECM-remodeling, and myofibroblast (Fig. S6A–C)—with myofibroblast/ECM-remodeling fibroblasts enriched in T and inflammatory fibroblasts peaking at L. Endothelial cells partitioned into sinusoidal, tip/angiogenic, and stalk-like states with region-dependent redistribution (Fig. S6D–F).

(A) UMAP of fibroblasts with subtype annotations: portal/periportal-like

(PDGFRA$^+$/THY1$^+$), inflammatory/chemokine-rich (CXCL14$^+$/CFD$^+$/CXCL12$^+$), ECM-remodeling (COL1A1$^+$/COL3A1$^+$/MMPs), and myofibroblast (ACTA2$^+$/TAGLN$^+$/MYL9$^+$).

(B) Marker expression (dot/heatmap) supporting fibroblast subtype identities.

(C) Regional proportions of fibroblast subtypes across N, L, and T (per-patient stacked bars); myofibroblast and ECM-remodeling fibroblasts accumulate in T, whereas inflammatory fibroblasts peak at L (two-sided Fisher's exact with BH-FDR).

(D) UMAP of endothelial cells subtyped into sinusoidal (CLEC4G$^+$/STAB2$^+$/LYVE1$^+$), tip/angiogenic (KDR$^+$/DLL4$^+$/APLN$^+$/ESM1$^+$), and stalk/capillary-like (PECAM1$^+$/KDR$^+$/KLF2^low) states.

(E) Endothelial marker heatmap validating subtype calls.

(F) Regional proportions of endothelial subtypes across N/L/T (per-patient). Tip/angiogenic ECs are enriched in T, sinusoidal ECs dominate N, with stalk-like ECs intermediate in L (Fisher's exact, BH-FDR).

Abbreviations: N, normal; L, leading edge; T, tumor; EC, endothelial cell; BH-FDR, Benjamini–Hochberg false discovery rate.
